# Supplementary figures and images for: Comparison of ultrasound and dynamic MRI for the measurement of diaphragmatic excursion: A prospective single-center study
Source: PLoS One. 2025 Feb 21;20(2):e0318717. doi: 10.1371/journal.pone.0318717 (PMC12005672; doi:10.1371/journal.pone.0318717)

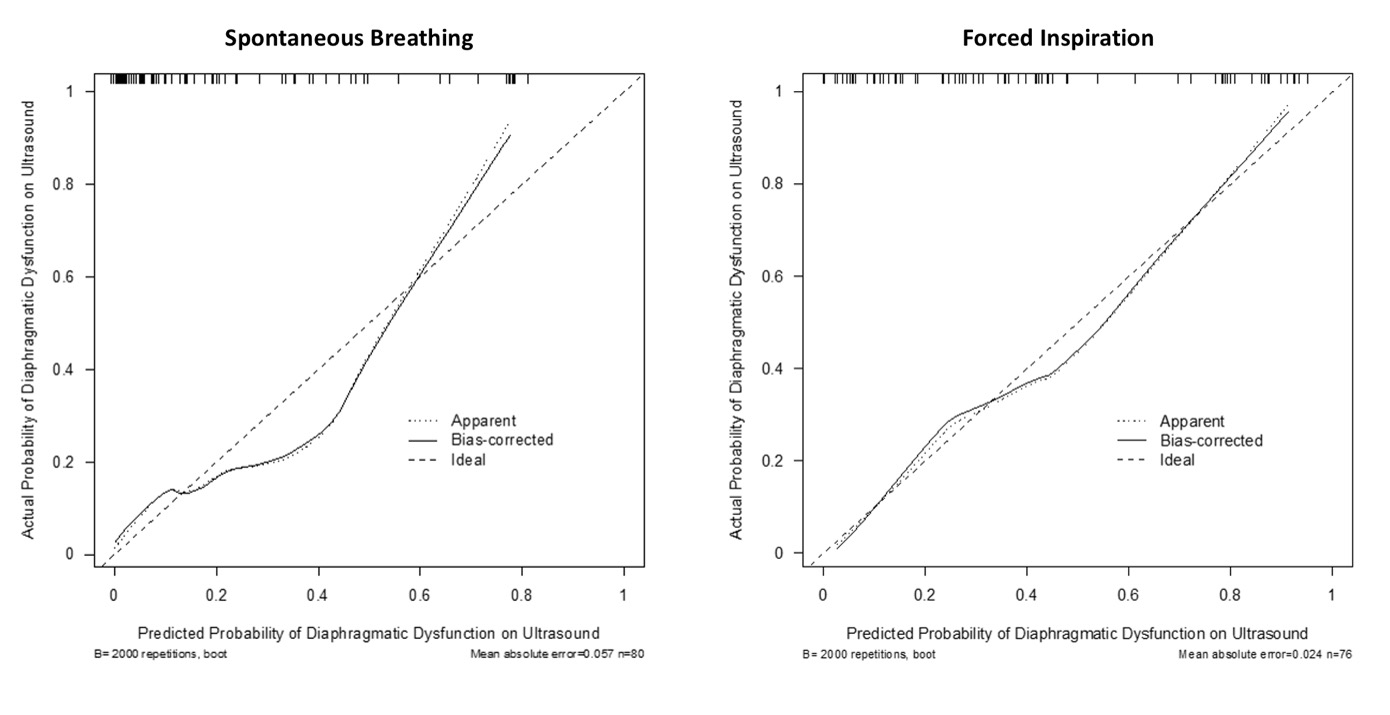

Supplement: S1 Fig — (TIF) [file pone.0318717.s001.tif]

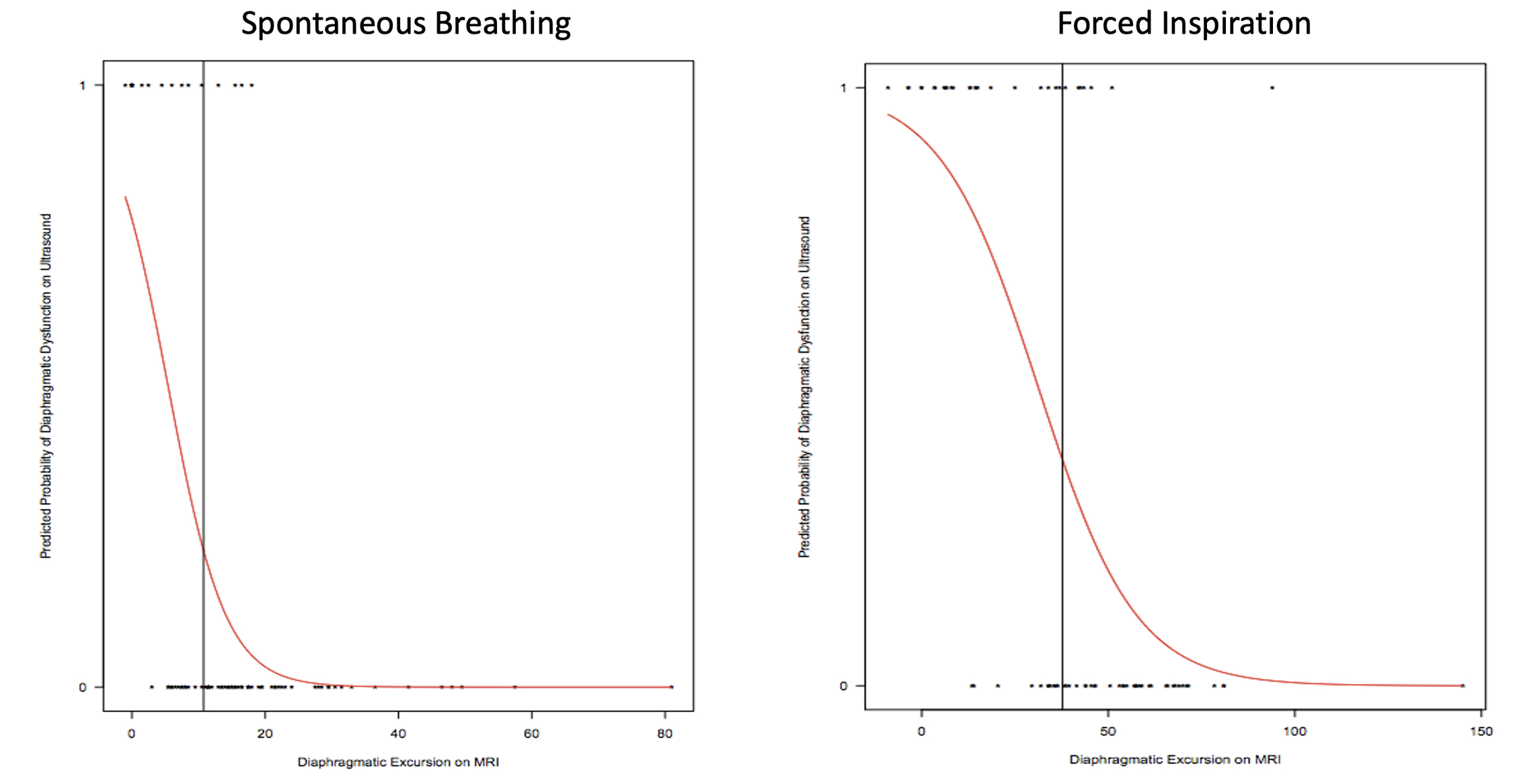

Supplement: S2 Fig — (TIF) [file pone.0318717.s002.tif]
